# Current Understanding on the Genetic Basis of Key Metabolic Disorders: A Review

**DOI:** 10.3390/biology11091308

**Published:** 2022-09-02

**Authors:** Kenneth Francis Rodrigues, Wilson Thau Lym Yong, Md. Safiul Alam Bhuiyan, Shafiquzzaman Siddiquee, Muhammad Dawood Shah, Balu Alagar Venmathi Maran

**Affiliations:** 1 Biotechnology Research Institute, Universiti Malaysia Sabah, Kota Kinabalu 88400, Malaysia; 2 Borneo Marine Research Institute, Universiti Malaysia Sabah, Kota Kinabalu 88400, Malaysia

**Keywords:** bioinformatics, biomarkers, epigenetics, genetic modifications, genome editing, genome wide association studies

## Abstract

**Simple Summary:**

Metabolic disorders (MD) are a challenge to healthcare systems; the emergence of the modern socio-economic system has led to a profound change in lifestyles in terms of dietary habits, exercise regimens, and behavior, all of which complement the genetic factors associated with MD. Diabetes Mellitus and Familial hypercholesterolemia are two of the 14 most widely researched MD, as they pose the greatest challenge to the public healthcare system and have an impact on productivity and the economy. Research findings have led to the development of new therapeutic molecules for the mitigation of MD as well as the invention of experimental strategies, which target the genes themselves via gene editing and RNA interference. Although these approaches may herald the emergence of a new toolbox to treat MD, the current therapeutic approaches still heavily depend on substrate reduction, dietary restrictions based on genetic factors, exercise, and the maintenance of good mental health. The development of orphan drugs for the less common MD such as Krabbe, Farber, Fabry, and Gaucher diseases, remains in its infancy, owing to the lack of investment in research and development, and this has driven the development of personalized therapeutics based on gene silencing and related technologies.

**Abstract:**

Advances in data acquisition via high resolution genomic, transcriptomic, proteomic and metabolomic platforms have driven the discovery of the underlying factors associated with metabolic disorders (MD) and led to interventions that target the underlying genetic causes as well as lifestyle changes and dietary regulation. The review focuses on fourteen of the most widely studied inherited MD, which are familial hypercholesterolemia, Gaucher disease, Hunter syndrome, Krabbe disease, Maple syrup urine disease, Metachromatic leukodystrophy, Mitochondrial encephalopathy lactic acidosis stroke-like episodes (MELAS), Niemann-Pick disease, Phenylketonuria (PKU), Porphyria, Tay-Sachs disease, Wilson’s disease, Familial hypertriglyceridemia (F-HTG) and Galactosemia based on genome wide association studies, epigenetic factors, transcript regulation, post-translational genetic modifications and biomarker discovery through metabolomic studies. We will delve into the current approaches being undertaken to analyze metadata using bioinformatic approaches and the emerging interventions using genome editing platforms as applied to animal models.

## 1. Introduction

Metabolic diseases (MD) are becoming increasingly prevalent across genetically diverse populations across the world [1] and they pose an increasing economic burden on public health systems in both the developing countries located in Africa [2], South East Asia [3] and developed countries such as Japan [4] and Australia [5]. MD also poses a significant challenge to the mental health of patients [6,7], which in turn has an impact on productivity and the quality of life. Mitigating the long-term effects of MD has led to the development of a wide range of interventions that rely on the high resolution of modern molecular tools to detect and diagnose MDs by examining the leading and lagging indicators in patients. Our current understanding of the molecular mechanisms of disease has led researchers on a path to investigate the genomic, transcriptomic, and metabolomic as well as environmental, dietary and microbial factors that can lead to the development of MD. Molecular approaches, which range from genetic screening for the early detection and diagnosis of genes linked to MDs [8] in newborns [9] and the development of standards for genetic screening in the case of specific MD [10]. The ongoing research has also delved into the role of epigenetics in gene regulation [11,12], the role of the transcriptome, encompassing variations in RNA transcripts in MD [13] and the role of regulatory RNA [14].

The development of high-resolution proteomic screening has led to advances in the development of biomarkers based on metabolomic profiling [15] and the detection of temporal events, which can be implicated in the development of MD [16]. All of these developments have led to the synthesis of information derived from multi-omic studies [17,18,19] with the objective of arriving at a definitive and comprehensive overview of the regulatory factors involved in the development of MDs. The rapid advances of microbial metagenomics [20,21,22] and the correlation between microbial metabolites secreted in the gut and MDs [23,24,25,26] have led researchers to propose novel alternative therapies including the use of bacterial inoculum as dietary supplements [27] as well as dietary supplements that favor the establishment of a healthy gut microbiome [28], has led to the dawn of a new era in the management of diet and microbial populations in order to ameliorate the deleterious effects of MDs in a sustainable manner. Looking beyond diagnostics and dietary interventions, the discovery and development of models for genetic modification have offered new hope to patients with inherited MDs. The recent development of CRISPR-Cas13 [29]-based genome editing platforms has emerged as one of the frontline technologies in the battle against MD. The targeting of specific RNA is using CRISPR-Cas13 [30] in specific cells and tissues [31] using novel methods for the delivery of nucleic acids [32] to their intended targets. CRISPR-based therapeutics still face many challenges, primarily due to their lack of precision in modifying specific genomic loci [33] and off-target activation [34]; however, these will be mitigated as genomic data continues to provide a high resolution map of the genome.

The rapid development of next generation sequencing (NGS) technologies and their deployment across diverse populations has provided clinicians with one of the most valuable resources for the early detection and diagnosis of genes associated with specific MD. Genome wide association studies (GWAS) have further reinforced the relationship be-tween specific gene polymorphisms and the onset, progression and severity of inherited genetic disorders [35]. Interventions based on the genetic data must be made with caution and in accordance with specific guidelines that have been developed over multiple cycles of testing and assessment. The clinical front has witnessed the development and release of multiple contemporary therapeutic approaches for the management of MD. The high developmental cost associated with orphan drugs to treat rare metabolic disorders [36,37] and their limited usage [38] across the world [39] due to the high cost [40,41,42,43] has led to the proposition of novel approaches such as the development of personalized therapeutics using RNA interference (RNAi)-based platforms [44]. Concerns regarding the inherent instability of the RNA molecule and difficulties in the industrial manufacture initially resulted in the idea of employing messenger RNA (mRNA) as a therapeutic molecule with skepticism. These challenges are now being addressed, thanks to technological advancements and the development of RNA production technology that relies on the enzyme RNA polymerase [45] for the in vitro production of high-quality molecules with high fidelity.

As a result, the potential of mRNA for the development of novel revolutionary therapies is being increasingly recognized, with applications in immunotherapy, regenerative medicine, vaccination, and gene editing all being considered [46]. RNA therapeutics have emerged as one of the most promising areas for the development of personalized therapeutics for treating MDs, as is evidenced by a significant increase in the number of research projects focusing on RNA-based platforms [47], and synthetic antisense oligonucleotides (ASO) have led to the development of the first RNA-targeted drug Nusinersen [48], which has been touted as one of the first commercially successful ASO in the treatment of spinal muscular atrophy [49]. Dietary interventions in the form of substrate reduction therapy (SRT) have demonstrated promising results in the case of lysosomal storage diseases such as GD, wherein SRT in clinical settings was found to adequately reduce the visceral management of GD [50] without a direct impact on brain disease, as evinced in a murine cell line-derived neural model [51]. Lucerastat, an iminosugar [52], is an inhibitor of glucosylceramide synthase, has the potential for substrate reduction therapy in glycosphingolipid storage disorders such as Fabry disease [52].

**Figure 1:**
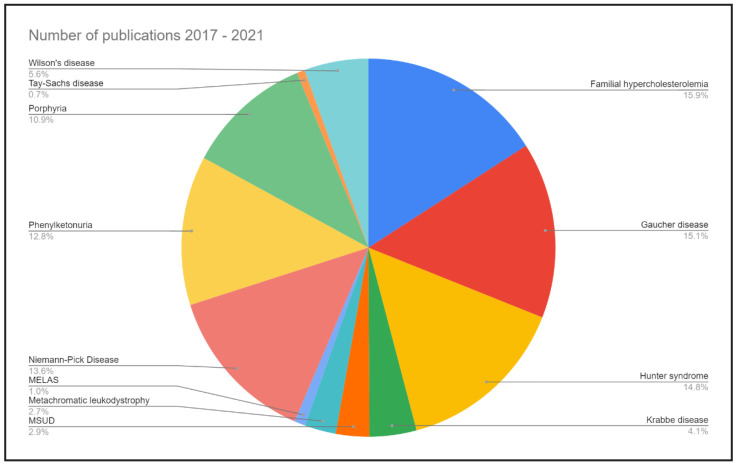
Research trends over the five-year period (2017–2021) reveal the extent of research being published in the 12 reviewed MD. The neglected MD are Tay-Sachs disease and MELAS. DM was the most extensively researched topic, with 129,829 publications (data not included in the graph in order to retain scale).

A PubMed search using specific search terms in the title over the past 5 years (2017 to 2021) indicates that out of a total of 7045 titles that were retrieved, a majority focused on Familial hypercholesterolemia, Gaucher disease, Hunter disease and Niemann-Pick disease. The lowest number of publications was related to Tay-Sachs disease. The most highly researched topic was DM with 129,829 publications (Figure 1).

## 2. Diabetes Mellitus (DM)

The most common MD, which has been reported globally is diabetes mellitus (DM) [53] and mapping of global trends has indicated an increase in research related to this non-communicable disease (NCD) [54]. It is projected that the current reported caseload of 463 million people living with diabetes will increase steadily over the next three decades [55], prompting the need for interventions at multiple levels including recommending changes in lifestyle [56], management of gut microflora [57,58], the implantation of stem cells [59] and genome editing using CRISPR-based platforms [60]. The current COVID-19 pandemic has highlighted the fact that patients with comorbidities such as diabetes have an increased SARS-CoV-2 susceptibility [61] and further complications, which include insulin resistance and lipid metabolic dysregulation [62]. The progression of the metabolic syndrome (MS) from the high-income countries in the Western world to the less affluent countries has highlighted the importance of regulation of dietary intake and an active lifestyle as an avenue to the management of this NCD. MS comes at a significant cost to the global economy, which is estimated to be in the billions of dollars when taking into account the loss of productivity resulting from morbidity [63]. Around 250 genomic loci have been linked with type 2 diabetes propensities in genome-wide association studies, with evidence for causative variants and genes developing for several of these regions. The integration of multidimensional data for diabetes-related intermediate phenotypes, detailed genomic annotations, functional experiments, and now multiomic molecular features has aided understanding of the underlying mechanisms, including the interplay between cellular failure, insulin sensitivity, appetite regulation, and adipose storage. The significance and need for a wide genetic approach to this global disease has been demonstrated by studies in a variety of ethnic groups and examples from population isolates. Some of the Eurocentric bias could be addressed through trans ethnic discovery efforts and large-scale biobanks of diverse populations and ancestries.

Despite rapid advances in understanding the highly polygenic architecture of type 2 diabetes, which is dominated by common alleles with small, cumulative effects on disease risk, these insights have been of limited clinical utility in terms of disease prediction or prevention, and have contributed only modestly to subtype classification or stratified treatment approaches. Exome or genome sequencing in big biobanks could benefit from the establishment of academia-industry collaborations, with implications for the future of genomics-focused research [64]. The major histocompatibility complex (MHC) is situated on chromosome 6p21 and comprises critical immune response regulators, including human leucocyte antigen (HLA) genes, as well as non-immunological genes [65]. GWAS has offered a plethora of knowledge on the genetic basis of T1D, and over 50 loci have been related with this condition, in addition to the genes found using candidate gene techniques [66]. The HLA region on 6p21 accounts for around 50% of the familial aggregation of T1D and has been linked to T1D for over 40 years. The strongest links are with HLA DR and DQ. HLA DR and DQ are antigen-presenting cell surface receptors. Alpha-beta heterodimers DR and DQ. DRA loci encode the DR alpha chain, while DRB loci encode the DR beta chain. The DQA1 and DQB1 loci encode the DQ molecule’s alpha and beta chains, respectively. The DR and DQ loci are highly connected, as are other HLA loci. The insulin gene (INS) is linked to T1D more than the HLA region. There are three main insulin VNTRs on chromosome 11p15. Homozygosity for class I, these insulin polymorphisms likely influence the development of immune tolerance to insulin.

## 3. Familial Hypercholesterolemia (FH)

Familial hypercholesterolemia (FH) is an inherited metabolic disorder that is characterized by deficiencies in the clearance of low-density lipoprotein (LDL) molecules from the liver, which, if left untreated, can lead to hypercholesterolemia. The FH phenotype has been encountered in approximately 1 of 300 individuals [67]. There has been an increase in scientific research in FH, which has been spearheaded by pharmaceutical companies in the areas of detection, diagnosis and treatment and 12 drugs have been approved and released in Europe, Japan, the United States and other regions of the world [68]. Implementation strategies for the treatment of FH are key to improving the long-term care of patients and a pertinent guideline has been developed by the Expert Recommendations for Implementing Change (ERIC), which is aimed at driving translational research [69]. The development of new experimental molecules such as proprotein convertase subtil-isin/kexin type 9 inhibitors (PCSK9i) [70] and association therapy based on monoclonal antibodies (mAbs) targeting PCSK9, evolocumab and alirocumab for treatment of patients diagnosed with heterozygous FH (HeFH) or both in the most severe homozygous FH (HoFH) has offered promising results in clinical trials [71]. The primary causes of FH are pathogenic mutations in the genes encoding the LDL receptor (LDLR), its ligand the apolipoprotein B (APOB) or Proprotein Convertase Subtilisin/Kexin Type 9 (PCSK9) (PCSK9). Mutations in the genes expressing apolipoprotein E (APOE) and the signal-transducing adaptor family member 1 are rarer causes (STAP1).

The genetics of FH is very complex due to a high heterogeneity, the presence of variation clusters and clinical variability. In fact, a significant diversity was detected among individuals with the same familial status: an overlap of LDL-cholesterol levels was reported across heterozygous patients (HeFH) and homozygous FH patients, and even some HeFH exhibited a similar lipid profile. A proper pathogenicity assessment is the first step in correctly defining the genetic status and identifying the variations that cause FH. HeFH patients with distinct variant types (null or faulty) or variations in different impacted genes showed several phenotypic differences. Patients with a null variant in the LDLR gene had greater LDL cholesterol levels and were more likely to develop coronary artery disease than those with a faulty variant. Pathogenic mutations in multiple lipid-related genes have been discovered in FH patients, serving as both modifying (worsening the symptom) and confounding factors (requiring a differential diagnosis to distinguish from FH) [72]. Only 1.7–2.5 percent of participants with an LDL-C > 4.9 mmol/L (190 mg/dL) had an FH-causing mutation identified in two large population-based trials involving 76,751 people. DNA sequencing validates the diagnosis of FH in unselected patients whose only criterion is an elevated LDL-C, but it has a low yield in patients whose only criterion is an elevated LDL-C. Treatment initiation and adherence are both improved. Depending on the degree of the DNA mutation, patients with an FH-causing variation have a 4.4- to 6.8-fold greater risk of ASCVD compared to controls [73]. Only medications that inhibit cholesteryl ester transfer protein (CETP), angiopoietin-related protein 3 (ANGPTL3), and apolipoprotein C-III (apoC-III) have lately been explored in clinical trials, despite the fact that about 80 genes are linked to hypercholesterolemia. CETP and ANGPTL3 inhibition reduced LDL cholesterol. Inhibition of ANGPTL3 showed the greatest effect and was even beneficial in patients with familial hypercholesterolemia. The impact of apoC-III inhibition on LDL cholesterol has yet to be determined [74]. Multiple alleles involved in low-density lipoprotein regulation could play a role in the development of familial hypercholesterolemia, particularly in patients with mutation-negative familial hypercholesterolemia. Multiple uncommon genetic variants contributed to more severe familial hypercholesterolemia in oligogenic familial hypercholesterolemia [75]. In a study of 487 FH patients, those who did not have mutations had considerably higher polygenic scores than those in the other groups. LDL-C values were markedly higher in oligogenic FH participants than in monogenic FH subjects. Additionally, the mutations had a considerable impact on sitosterol/lathosterol levels. Rare and detrimental mutations in ABCG5/ABCG8 play a significant role in imitating and intensifying the FH phenotype [76].

## 4. Gaucher Disease (GD)

Gaucher disease (GD) is an autosomal recessive genetic disease whose incidence varies between 0.4 and 5.8/100,000 inhabitants; GD can be attributed to a deficiency of the lysosomal enzyme, glucocerebrosidase, which leads to the accumulation of its substrate (glucosylceramide) in lysosomal macrophages [77]. Type-1 Gaucher disease is prevalent in European and North American populations and manifests as deformities in the viscera. Types 2 and 3 are also associated with neurological impairment, either severe in type 2 or variable in type 3 [78]. There have been many advances in the treatment of GD as a result of the pioneering work by Dr. Roscoe Brady [79], which included identification of the genetic factors and the synthesis of recombinant glucocerebrosidase [80]. Some of the major challenges that remain to be addressed in the case of GD are related to wide phenotypic diversity including non-neuronopathic, acute neuronopathic, and chronic neuronopathic manifestations. A review of the scientific literature related to GD revealed an increase in interest and research, with challenges in the areas of biomarker discovery for monitoring progression and a lack of personalized treatment regimens [81]. Gaucher disease (GD) is an autosomal recessive disorder caused by the deficiency of glucocerebrosidase, a lysosomal enzyme that catalyzes the hydrolysis of the glycolipid glucocerebroside to ceramide and glucose. Lysosomal storage of the substrate in cells of the reticuloendothelial system leads to multisystemic manifestations, including involvement of the liver, spleen, bone marrow, lungs, and nervous system.

Patients with GD have highly variable presentations and symptoms that, in many cases, do not correlate well with specific genotypes. Almost 300 unique mutations have been reported in the glucocerebrosidase gene (GBA), with a distribution that spans the gene. These include 203 missense mutations, 18 nonsense mutations, 36 small insertions or deletions that lead to either frameshifts or in-frame alterations, 14 splice junction mutations, and 13 complex alleles carrying two or more mutations in cis. Recombination events with a highly homologous pseudogene downstream of the GBA locus also have been identified, resulting from gene conversion, fusion, or duplication. In this review, we discuss the spectrum of GBA mutations and their distribution in the patient population, evolutionary conservation, clinical presentations, and how they may affect the structure and function of glucocerebrosidase [82]. Gaucher disease (GD) and Parkinson’s disease (PD) have been linked for nearly two decades. Mutations in the glucocerebrosidase gene (GBA) may result in GD, a condition in which glucosylceramide, the sphingolipid substrate of the glucocerebrosidase enzyme (GCase), accumulates in visceral organs, resulting in a variety of clinical symptoms. GBA mutations enhance the likelihood of Parkinson’s disease in the biallelic or heterozygous stage. GBA allele mutations are the most major genetic risk factor for idiopathic PD, accounting for between 5% and 20% of idiopathic PD cases, depending on ethnic origin [83]. A novel point mutation, g.12599C > A (c.999 + 242C > A), was detected deep in intron 7 of the GBA gene. This type of mutation has been previously described for other diseases, but this is the first time, as far as we know, that it has been described for GD. This mutation creates a new donor splice site leading to aberrant splicing and resulting in the insertion of the first 239 nt of intron 7 as a pseudoexon in the mRNA, creating a premature stop codon [84]. A study conducted in Iran detected six new mutations of the GBA gene among GD patients. Two mutations (p.L483P and p.N409S) were especially common among Iranians; this finding can be used in implementing screening programs and understanding the molecular basis of GD [85].

## 5. Mucopolysaccharidosis Type II (MPS II) (Hunter Syndrome)

Mucopolysaccharidosis type II (MPS II) (Hunter syndrome) is linked to the deficiency of the lysosomal hydrolase iduronate 2-sulphatase, which is a critical enzyme involved in the stepwise degradation of heparan and dermatan sulphate. MPS II was discovered almost a century ago, and is extremely rare with an incidence of 0.38 to 1.09 per 100,000 live male births and has been linked to the X chromosome [86]. Enzyme replacement therapy (ERT) is one of the options for patients diagnosed with the phenotype [87]; however, the inability of the intravenously administered enzymes to overcome the blood brain barrier renders ERT ineffective against progressive neurodegeneration and severe symptoms in the Central Nervous System as reported in patients with neuronopathic MPS [88]. Recent advances in the development of RNA technology have led to novel approaches such as the experimental production of human iduronate-2-sulfatase (IDS) using a Sendai virus vector [89].

## 6. Krabbe Disease (KD)

Krabbe disease (KD) is an autosomal recessive neurodegenerative disorder that manifests as the inability of the enzyme Galactosylceramidase to hydrolyse galactosylceramide and psychosine within lysosomes [90]. Insights into genetic aspects of KD have led to the development of diagnostic procedures [91]; however, there is a significant healthcare burden associated with the management of patients with KD [92] and current research efforts have been directed toward exploring gene therapy using animal models [93]. Branched-chain α-ketoacid dehydrogenase deficiency (maple syrup urine disease) (MSUD) is an inborn error of metabolism caused by alterations in the branched-chain α-ketoacid dehydrogenase complex, which results in elevated levels of branched-chain amino acids (BCAAs) in plasma, α-ketoacids in the urine the secretion of the biomarker alloisoleucine [94], MSUD has also been associated with neurological [95] and neuro-psychiatric disorders, which in turn pose therapeutic and diagnostic problems [96]. Current approaches to the management of MSUD rely on nutrition management [97] and a domino liver transplantation using a phenotypically normal explant from a selected recipient and a donor graft from another patient [98], which compensates by the systemic presence of a sufficient enzyme [99].

## 7. Metachromatic Leukodystrophy

Metachromatic leukodystrophy is a demyelinating, autosomal recessive hereditary leukodystrophy and LSD caused by an inborn metabolic error in the lysosomal enzyme arylsulfatase A. This causes a buildup of sulfatides, which causes the CNS/PNS myelin sheaths to become dysfunctional and their gradual degradation. It can manifest in other organs as well, such as the kidneys, testes, and gallbladder. It can be categorized de-pending on the disease’s onset age and clinical symptoms. There is a gradual decline of neurodevelopment and neurocognitive function in all forms of the disease [100]. As with X-linked adrenoleukodystrophy, a universal screening panel is recommended. For certain leukodystrophies, stem cell therapies have become standard of care. However, the hazards associated with transplanting remain significant, and the results are not always adequate. Ex vivo gene therapy, which involves infecting autologous hematopoietic stem cells with lentiviral vectors, avoids some, but not all, of the risks associated with traditional transplantation and has recently been demonstrated to be safe and effective in clinical studies of X-linked adrenoleukodystrophy and metachromatic leukodystrophy. For many monogenetic pediatric neurological illnesses, the direct infusion of adeno-associated virus vectors has emerged as a safer alternative to gene therapy. Adeno-associated viral gene therapy has been found to be safe and effective in leukodystrophies in a number of preclinical studies, allowing for broader access treatment for Canavan disease prior to the start of a clinical trial [38].

## 8. Mitochondrial Encephalopathy with Lactic Acidosis and Stroke-like Episodes (MELAS) Syndrome

Stroke-like episodes (SLEs) are a defining feature of mitochondrial encephalopathy with lactic acidosis and stroke-like episode (MELAS) syndrome, although they can also happen in other conditions [101]. Errors in the translation of nuclear-encoded proteins by the mitochondrial protein translation system lead to the early-onset and deficiency of one or more oxidative phosphorylation (OXPHOS) complexes in specific tissues [102]. Data has emerged to encourage the consideration of utilizing additional therapeutic agents and the withdrawal of numerous previously used medicines since the publication of Mitochondrial Medicine Society recommendations for mitochondrial medicine therapy management in 2009. Preclinical animal modeling studies demonstrated that vitamin C was ineffective as an antioxidant for primary mitochondrial dysfunction, but that vitamin E and N-acetylcysteine were effective. Clinical evidence suggests that L-carnitine may hasten the progression of atherosclerosis. More data supporting the clinical use of L-arginine as a prophylaxis against or acute treatment for metabolic strokes in people with mitochondrial encephalopathy with lactic acidosis and stroke-like episodes (MELAS) syndrome and Leigh syndrome has come from long-term follow-up. In addition, various precision medicines for particular genetic causes and/or shared clinical manifestations of primary mitochondrial disease have been discovered [103].

## 9. Niemann-Pick Disease (NPD)

Niemann-Pick disease (NPD) is caused by an acid sphingomyelinase deficiency (ASMD), a lysosomal storage disorder characterized by the hydrolysis of sphingomyelin (SM) to ceramide and phosphocholine. As a result, macrophage lysosomes accumulate SM and its precursor lipids. These lipid-laden macrophages aggregating in the liver, spleen, lungs, and brain elicit hepatosplenomegaly, cytopenias, lung disease, and neurologic symptoms [104]. Despite significant discoveries, Niemann-Pick type C (NPC) is an example of a disease that lacks a cure. Defects in NPC1 or NPC2 cause this rare, deadly, autosomal-recessive condition. These widely expressed proteins assist cholesterol in leaving the endosomal-lysosomal pathway. Patients with a wide variety of disease onset, neurovisceral symptoms, and life spans have abnormal lipid buildup due to either malfunction [105]. The body’s natural cholesterol scavenging particle, the high-density lipoprotein (HDL), has been leveraged by researchers to produce new therapies for this illness [106].

## 10. Phenylketonuria (PKU)

Phenylketonuria (PKU) is an autosomal recessive phenylalanine metabolism mistake due to defects in the enzyme phenylalanine hydroxylase, which converts phenylalanine to tyrosine. If left untreated, PKU causes elevated phenylalanine concentrations in the blood and brain, resulting in severe intellectual impairment, seizures, and behavioral issues [107]. Neonatal screening and dietary therapy with a low-phenylalanine diet have improved the prognosis for phenylketonuria (PKU). This treatment must be followed for the rest of one’s life, which causes serious compliance issues. Sapropterin (or BH4) has been demonstrated to benefit a smaller percentage of patients who respond to it [108]. In a single study, those who maintained the diet had a considerably higher Intelligence Quotient than those who discontinued the diet, MD after 12 months 5.00. (95 percent CI 0.40 to 9.60) [109]. Management of the gut microbiota is an alternative therapeutic strategy. Dietary composition is of great interest in inherited errors of metabolism (IEMs) because of its function in microbiota modulation, and it may provide an intriguing treatment target. Because part of the therapeutic intervention in IEMs, such as phenylketonuria (PKU), is based on chronic or life-long adjusted dietary regimens, significant fluctuations in microbial diversity or relative abundance have been documented [110]. The murine model has been applied to test a range of possible gene-based therapies using the recombinant adeno-associated viral and non-viral DNA vectors-based gene complementation, and genome editing using recombinant Adenovirus vectors, all of which are designed for tissue specific expression in the mouse liver and skeletal muscle [111].

## 11. Hereditary Porphyrias

A collection of eight metabolic diseases of the heme biosynthesis pathway known as hereditary porphyrias are characterized by acute neurovisceral symptoms, skin lesions, or both. Each porphyria is caused by aberrant enzyme action, resulting in a particular buildup of heme precursors [112]. The appropriate concentrations of vitamins and minerals in the tissues are required for certain enzymes involved in heme production. Furthermore, micronutrients required for the biosynthesis of succinyl CoA and other intermediates in the Krebs (TCA) cycle are also critical for heme metabolism indirectly [113] Givosiran, a small interfering RNA (siRNA)-based therapeutic developed for the treatment of acute intermittent porphyria (AIP), a condition that is characterized by life-threatening acute neurovisceral episodes, was approved by the US Food and Drug Administration (FDA) in November 2019 [114]. Patients with acute intermittent porphyria who were administered givosiran had a reduced rate of porphyria attacks and better results for a variety of other disease symptoms than those who were given a placebo. A higher prevalence of hepatic and renal side effects followed the enhanced efficacy [115].

Tay-Sachs disease (TSD) is a progressive, fatal neurodegenerative disorder caused by a lack of the enzyme hexosaminidase-A, which results in the accumulation of GM2 gangliosides. The disease is divided into three categories based on the age of onset: infantile, juvenile, and adult. The limited clinical symptoms and nonspecific biochemical data contribute to the difficulty in the early diagnosis of TSD. Accurate diagnosis is critical for proper management and the reduction of disease-related consequences [116]. TSD treatment is currently focused on symptom alleviation and, in the case of the late-onset variant, delaying progression. There have also been clinical reports of miglustat and bone marrow or hematopoietic stem cell transplantation being used to lower the concentration of substrate. Gene therapy has been explored using adeno- or adeno-associated viruses as vectors for delivering cDNA encoding HexA subunit genes. The efficacy of this technique has been tested in HexA deficient mice and Jacob sheep, in which Tay-Sachs disease develops naturally and has the same clinical characteristics as in humans [117]. Wilson disease (WD) is the result of a metabolic error in copper metabolism that is hereditary. As a result of the improper management of copper, hepatocytes become toxic, and copper levels in the blood rise. Other organs, particularly the central nervous system, assimilate copper. WD affects people of all ages, usually between the ages of 5 and 35, although it now affects people of all ages. Ref. [118] WD is diagnosed utilizing diagnostic methods that include clinical symptoms and indicators, copper metabolism measurements, and ATP7B DNA analysis. Chelation therapy and zinc salts are two treatments that reverse copper overload through separate methods. A study investigating the association between the bioavailability of copper and atherosclerosis determined that the baseline level of copper can be extrapolated as an indicator for the early detection of atherosclerosis [119] In some circumstances, liver transplantation is also recommended. Clinical trials for new drugs, such as tetrathiomolybdate salts, are now underway, and animal models are being used to evaluate genetic treatments [120].

## 12. Familial Hypertriglyceridemia

Familial hypertriglyceridemia (F-HTG) is an autosomal dominant genetic disorder which is characterized by the overproduction of very low density lipoprotein (VLDL) from the liver and has been associated with the lipoprotein lipase gene (LPL) [121]. More than 200 mutations have been documented within the 30 kb LPL gene, which consists of 10 exons and encodes a protein of 475 amino acids with a 27 amino acid signal peptide, with only a limited number having been evaluated for pathogenesis [122]. A study conducted in Oman in a consanguine population revealed an association between F-HTG and alteration in multiple genes, which included LPL as well as APOC2, APOA5, GPIHPB1, and LMF1, implying that the genetic disorder is polygenic [123]; this finding has been supported by evidence from China, which indicates that the severity of F-HTG is digenic [124] and is likely to involve several molecular mechanisms, including splice-site variation [125]. The treatment of F-HTG focuses on the reduction of low-density lipoprotein (LDL) cholesterol levels, followed by management of non–high-density lipoprotein cholesterol levels; this can be achieved by changes in lifestyle and dietary modifications [126] or by the administration of statins such as Atorvastatin, Lovastatin, Fluvastatin, Pravastatin Rosuvastatin and Simvastatin [127]. Fibrates [128], Niacin [129] and Fish oil [126,130,131], The development of alternatives to statins, which include Evinacumab, the angiotensin-like 2 monoclonal antibody [132], which has shown adequate safety in trials [133]. The resurgence of approval for monoclonal antibody treatments for rare disorders [134] and gene therapy in animal models [135] will set the direction for the advancement of therapeutics for the management of this emerging disorder.

## 13. Galactosemia

Galactosemia is a MD that is characterized by the inability to metabolize galactose. The genetic basis for this MD can be linked to the GALT gene (galactose-1-phosphate uridylyltransferase). Recessive mutations in the enzyme lead to the accumulation of Galactose 1-phosphate, which in turn, inhibits cellular metabolism leading to cellular toxicity. More than 200 mutations in the GALT gene have been linked to the MD [136], of which two have been reported in European and African lineages. A missense mutation presents itself as mild to severe cases of galactosemia of which four mutations have been identified as potential candidates for gene therapy [137]. A less severe presentation of galactosemia is associated with the Duarte variant [138]. Recently, genome wide association studies conducted in Argentina [139] identified 14 different mutations among 72 unrelated alleles and similar studies conducted in Sweden [140], Greece [141], Korea [142], Turkey [143], Lithuania [144] and India [145] have reported mutations that are confined to specific regions and populations. Current approaches for the treatment of galactosemia include neonatal screening [146] followed by dietary management via the reduction or substitution of galactose and lactose; however, long-term therapy required additional interventions such as molecular chaperones to correct the misfolded [147] GALT enzyme [148] and the reliance on animal models to characterize the genetic association between GALT mutations and galactosemia [149]. Replacement of the GALT gene in the murine model [150] has shown promise, and the recombinant adeno-associated virus-mediated gene therapy [151] has proven to be effective in human cell lines. The current trend is indicative of an increasing reliance on gene replacement as a method for the mitigation of this MD (Table 1).

## 14. Conclusions

Metabolic diseases are having an increasing impact on public health services across the developed and developing world as a result of the transition in lifestyles and dietary patterns. The collective impact of these diseases is reflected in the economic impact as healthcare systems struggle to cater to the rise in patients with MDs and the associated decrease in productivity. Advances in metabolomics and genome sequencing have led to the discovery of associations between genes and MDs. The development of novel approaches to the treatment of MDs by genetic interventions such as gene therapy and RNA interference offers a lifeline to patients with existing conditions. However, the primary approach to address should be founded upon interventions in lifestyle and dietary changes for the long-term sustainability of the public health system.

## Figures and Tables

**Table 1:** The molecular basis for metabolic disorders (MDs) can be attributed to a diversity of biological processes at the genomic, transcriptomic, and proteomic levels. Recent studies have reported that most MDs can be attributed to multiple genes, epigenetic regulation, transcript processing, and post translational modifications.

No	Metabolic Disorder	The Molecular Basis for the MD
1	Diabetes mellitus	Epigenetic mechanisms [11], long non-coding RNAs [13], microbiome [22,23,24,25,26,27,28,55,58]
2	Familial hypercholesterolemia (FH)	Genes encoding the LDL receptor (LDLR), apolipoprotein B (APOB) Proprotein Convertase Subtilisin/Kexin Type 9 (PCSK9), apolipoprotein E (APOE), signal-transducing adaptor family member 1 (STAP1) [72]
3	Gaucher disease	GBA gene [77,82,83,84]
4	Mucopolysaccharidosis type II	Iduronate-2-sulfatase (IDS) gene [152,153,154,155,156,157] IDS gene transcript regulation [158,159,160,161]
5	Krabbe disease (KD)	Galactosyl-Ceramidase (GALC) gene [162,163,164,165,166,167,168,169]
6	Metachromatic leukodystrophy	Arylsulfatase A (ARSA) gene [170,171,172,173,174,175,176,177,178], prosaposin (PSAP) gene [179,180,181,182]
7	Mitochondrial encephalopathy with lactic acidosis and stroke-like episodes (MELAS) syndrome	Mitochondrial rRNA transferase gene [101,102], nuclear and mitochondrial genes associated with MELAS [183,184], nuclear DNA polymerase gamma (POLG1) gene [185,186]
8	Niemann-Pick disease	Acid sphingomyelinase (SMPD1) gene [187,188,189] transcript regulation [190]
9	Phenylketonuria	Phenylalanine hydroxylase (PAH) gene [191,192,193,194,195,196,197,198,199,200,201], epigenetic regulation [202,203]
10	Porphyria	Uroporphyrinogen III synthase (UROS) gene [204,205,206]
11	Tay-Sachs disease	Beta-hexosaminidase A (HEXA) gene [207,208,209,210]
12	Wilson disease	ATPase copper transporting beta (ATP7B) gene [211,212,213,214,215]
13	Familial hypertriglyceridemia	Lipoprotein lipase (LPL) gene [216,217]
14	Galactosemia	Galactose-1-phosphate uridylyltransferase (GALT1) gene [137,218,219,220], Galactokinase 1 (GALK1) gene [221,222,223]

## Data Availability

Not applicable.
